# The Effects of Caffeine on Metabolomic Responses to Muscle Contraction in Rat Skeletal Muscle

**DOI:** 10.3390/nu11081819

**Published:** 2019-08-07

**Authors:** Satoshi Tsuda, Tatsuya Hayashi, Tatsuro Egawa

**Affiliations:** 1 Laboratory of Sports and Exercise Medicine, Graduate School of Human and Environmental Studies, Kyoto University, Kyoto 606-8501, Japan; 2 Laboratory of Health and Exercise Sciences, Graduate School of Human and Environmental Studies, Kyoto University, Kyoto 606-8501, Japan

**Keywords:** metabolome, skeletal muscle, exercise, muscle contraction, ergogenic effect

## Abstract

Exercise has beneficial effects on our health by stimulating metabolic activation of skeletal muscle contraction. Caffeine is a powerful metabolic stimulant in the skeletal muscle that has ergogenic effects, including enhanced muscle power output and endurance capacity. In the present study, we aim to characterize the metabolic signatures of contracting muscles with or without caffeine stimulation using liquid chromatography-mass spectrometry and capillary electrophoresis coupled to mass spectrometry. Isolated rat epitrochlearis muscle was incubated in the presence or absence or of 3 mM caffeine for 30 min. Electrical stimulation (ES) was used to induce tetanic contractions during the final 10 min of incubation. Principal component analysis and hierarchical clustering analysis detected 184 distinct metabolites across three experimental groups—basal, ES, and ES with caffeine (ES + C). Significance Analysis of Microarray identified a total of 50 metabolites with significant changes in expression, and 23 metabolites significantly changed between the ES and ES + C groups. Changes were observed in metabolite levels of various metabolic pathways, including the pentose phosphate, nucleotide synthesis, β-oxidation, tricarboxylic acid cycle, and amino acid metabolism. In particular, D-ribose 5-phosphate, IMP, O-acetylcarnitine, butyrylcarnitine, L-leucine, L-valine, and L-aspartate levels were higher in the ES + C group than in the ES group. These metabolic alterations induced by caffeine suggest that caffeine accelerates contraction-induced metabolic activations, thereby contributing to muscle endurance performance and exercise benefits to our health.

## 1. Introduction

Exercise contributes to health benefits by reducing the risk of several chronic diseases. These effects are partly attributed to metabolic alterations that occur in contracting skeletal muscles. Exercise enhances muscle insulin sensitivity and mitochondrial function by stimulating master metabolic regulators, such as 5′-AMP-activated protein kinase (AMPK), sirtuin 1, and peroxisome proliferator-activated receptor-γ co-activator 1α [1,2]. Recent evidence has suggested that secreted myokines from contracting skeletal muscles have positive effects on metabolic disorders [3]. Therefore, it is accepted that muscle contraction-induced metabolic activation is a key factor for maintaining normal physical function.

Caffeine is a powerful metabolic stimulant in the skeletal muscle. In vitro caffeine treatment of the skeletal muscle promotes insulin-independent glucose transport [4,5,6,7,8], fatty acid oxidation [8,9], Ca^2+^ release from the sarcoplasmic reticulum [10,11], and mitochondrial biogenesis [12]. We have recently demonstrated that caffeine increases the maximal capacity of contraction-stimulated AMPK activation and glucose transport in rat skeletal muscles [6]. Additionally, caffeine is thought to be an important contributor to ergogenic effects in humans. Meta-analyses have shown that caffeine intake has positive effects on muscle power output and endurance performance [13,14,15]. These findings suggest that caffeine accelerates muscle contraction-induced metabolic activation, thereby contributing to exercise benefits toward health promotion. However, there are no observations investigating the overall effects of caffeine on muscle contraction-induced metabolic activation in the skeletal muscle.

Metabolomic techniques are useful tools for the investigation of complex metabolic responses to muscle contraction [16,17]. In the present study, we aim to characterize the metabolic signatures of contracting muscles with or without caffeine stimulation, using liquid chromatography-mass spectrometry (LC-MS) and capillary electrophoresis coupled to mass spectrometry (CE-MS) analysis.

## 2. Materials and Methods

### 2.1. Animals

Male Sprague–Dawley rats (150–160 g) were purchased from Shimizu Breeding Laboratories (Kyoto, Japan). Rats were fed a standard diet (Certified Diet MF; Oriental Koubo, Tokyo, Japan) with *ad libitum* water and were subjected to overnight fasting before the experiments. All animal-related protocols were performed in accordance with the Guide for the Care and Use of Laboratory Animals as adopted and promulgated by the National Institutes of Health (Bethesda, MD, USA) and were approved by the Animal Use Committee at Kyoto University Graduate School of Human and Environmental Studies.

### 2.2. Muscle Treatment

Muscles were treated as previously described [6]. Rats were killed by cervical dislocation without anesthesia, and the epitrochlearis muscles were removed and mounted on to an incubation apparatus with the tension set to 0.5 g. The epitrochlearis muscle is composed predominantly of fast-twitch glycolytic fibers (60–65% fast-twitch white, 20% fast-twitch red, 15% slow-twitch red) [18], but also has higher oxidative potential than the other fast-twitch muscle [19]. Moreover, it is a small and thin muscle that is suitable for in vitro incubation study. The muscles were pre-incubated in alpha-minimum essential medium (21444-05, nacalai tesque, Kyoto, Japan) containing 1.0 g/L glucose supplemented with 1% penicillin/streptomycin for 40 min and then incubated in fresh medium in the presence or absence of 3 mM caffeine for 30 min. For tetanic contractions, the muscles were stimulated using an electric stimulator (SEN-3401; Nihon Koden, Tokyo, Japan) during the final 10 min of the incubation period (train rate, 1/min; train duration, 10 s; pulse rate, 100 Hz; pulse duration, 0.1 ms; voltage; 10 V). Basal muscles were pre-incubated and incubated without contraction and caffeine treatment. All media were continuously gassed with 95% O_2_/5% CO_2_ and maintained at 37 °C.

### 2.3. Metabolomic Analysis

Metabolomic analysis was performed by LSI Medience Corporation (Tokyo, Japan). In brief, the muscle samples (≥50 mg) were homogenized using beads and suspended into 1 mL distilled water. They were then mixed with methanol (2 mL) and chloroform (2 mL) for 10 min at room temperature. After centrifugation at 1000× *g* for 15 min, the supernatant was evaporated using nitrogen gas and dissolved with 10% acetonitrile aqueous solution (200 µL). After adding internal standards, the samples were subjected to both LC-MS and CE-MS. All peak positions (retention time and *m*/*z*) and areas were calculated using Markeranalysis (LSI Medience, Tokyo, Japan). All peak areas were aligned into one data sheet, and the errors of peak intensities were corrected using internal standards. Noise peaks were deleted after comparison with the peaks detected in blank samples. The metabolites were identified by comparing the retention times and *m*/*z* values with a standard dataset provided by LSI Medience Corporation.

### 2.4. Data Analysis

After applying autoscaling (mean-centered and divided by standard deviation of each variable), principal component analysis (PCA), significance analysis of microarray (SAM), hierarchical clustering analysis (HCA), and one-way ANOVA with Tukey’s multiple comparison test were performed using the web-based metabolomic data processing tool MetaboAnalyst 4.0 (http://www.metaboanalyst.ca, Xia Lab, McGill University, Montreal, Canada). In PCA, a score plot of the first and second principal components was generated. HCA was performed to exhibit simultaneous clustering of metabolites and samples by Euclidean distance using Ward’s method. Heat maps were generated by coloring the values of all data across their value ranges. False discovery rates (FDR) were calculated to reduce the risk of false positives by adjusted *p* values. FDR < 0.05 was defined as statistically significant.

## 3. Results and Discussion

### 3.1. Pattern Recognition of Metabolites

**Figure 1:**
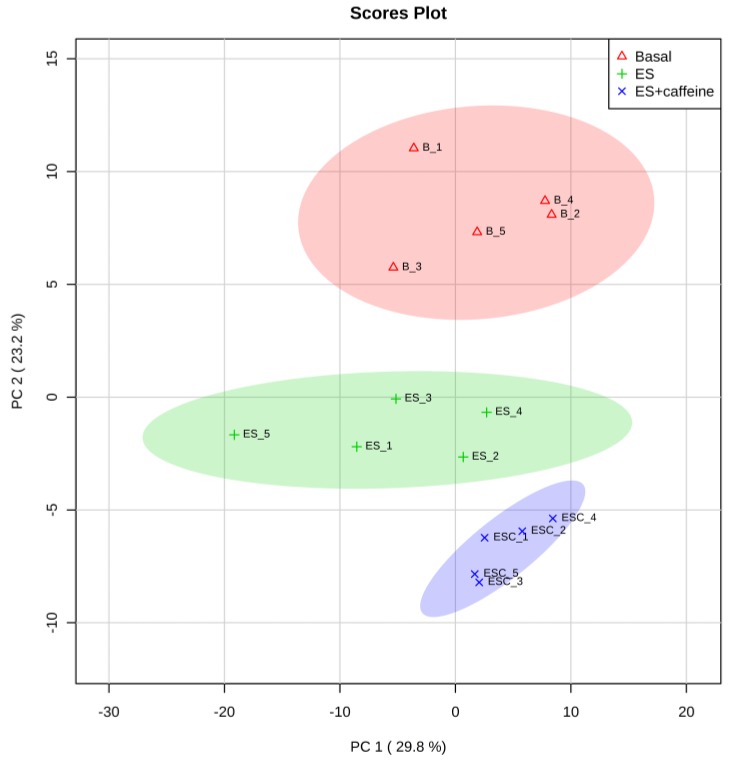
Principal component analysis (PCA) plot of the identified metabolites of the skeletal muscle of three groups, basal (B), electrical stimulation (ES), and electrical stimulation + caffeine (ESC). Principal components (PC1 and PC2) capture 53.0% of the variation in the dataset. The elliptic areas represent the 95% confidence regions.

Metabolomic analysis detected 184 metabolites by LC-MS and CE-MS (Table S1). PCA is a statistical procedure that is used for feature extraction. Using PCA on the detected 184 metabolites, three groups, i.e., basal, electrical stimulation (ES), and ES with caffeine (ES + C), were clearly distinguished on the principal component (PC) 2, although they were overlapped on PC1 (Figure 1). We previously demonstrated that ES in isolated rat skeletal muscles induces metabolic activation [20]. Likewise, the PCA results in this study indicated that ES-induced muscle contraction influences the metabolomic profile of the skeletal muscle. Furthermore, PCA plots in the ES + C group were more distant from basal than in the ES group (Figure 1), suggesting that caffeine accelerates ES-induced metabolic responses.

### 3.2. Discovery of Differentiating Metabolites

**Figure 2:**
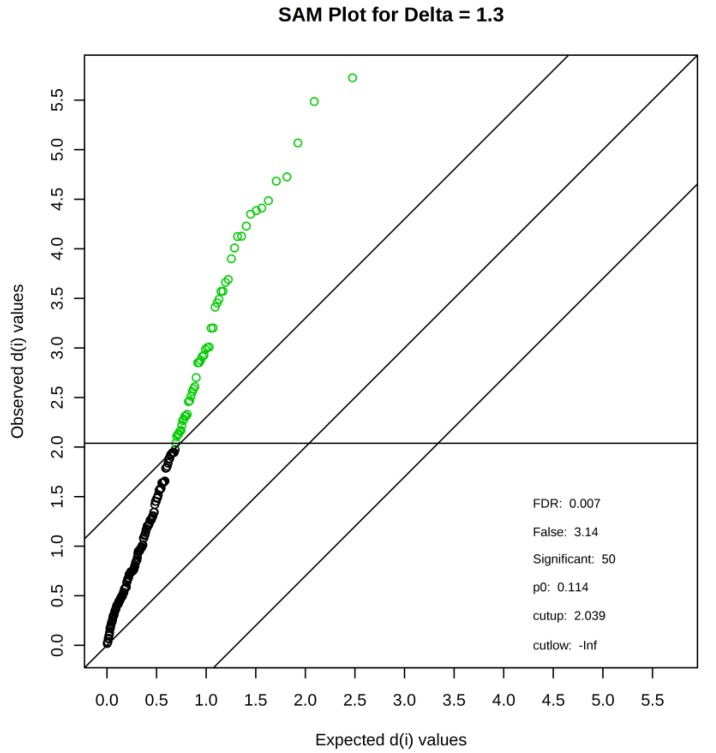
Identification of metabolites with significant changes in expression by significance analysis of microarray (SAM) among the three groups. The SAM plot is a scatter plot of the observed relative difference versus the expected relative difference. The solid diagonal line indicates where these two measures are the same. The dotted lines are drawn at a distance of delta from the solid line. The significant variables are highlighted in green and the details are shown in Table 1.

**Figure 3:**
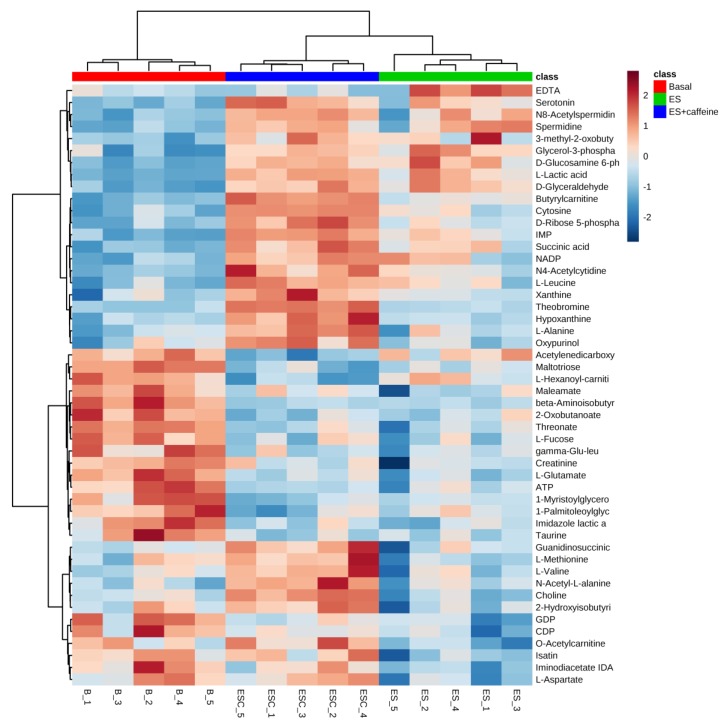
A heat map of hierarchical clustering analysis comparing the 50 different metabolites among groups. The heat map patterns among groups were distinguishable. The color red demonstrates that the relative content of metabolites is high and blue demonstrates that they are low.

**Figure 4:**
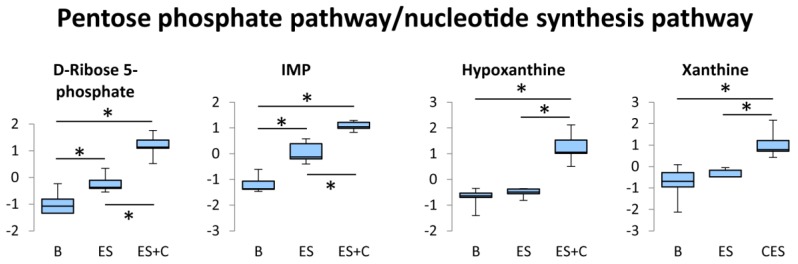
Box plots of the concentration variations of significantly altered metabolites in the pentose phosphate pathway/nucleotide synthesis pathway. Y axes are represented as normalized intensity. The boxes range from the 25% to the 75% percentiles. Medians are indicated by horizontal lines within each box. The ends of the whiskers represent the maximum and minimum of the data. * One-way ANOVA with Tukey’s post hoc test indicates a significant difference (false discovery rates (FDR) < 0.05) between groups.

**Figure 5:**
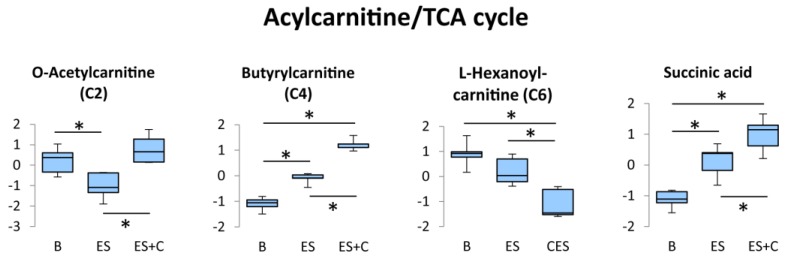
Box plots of the concentration variations of significantly altered metabolites in the Acylcarnitine/TCA cycle. * One-way ANOVA with Tukey’s post hoc test indicates a significant difference (FDR < 0.05) between groups.

**Figure 6:**
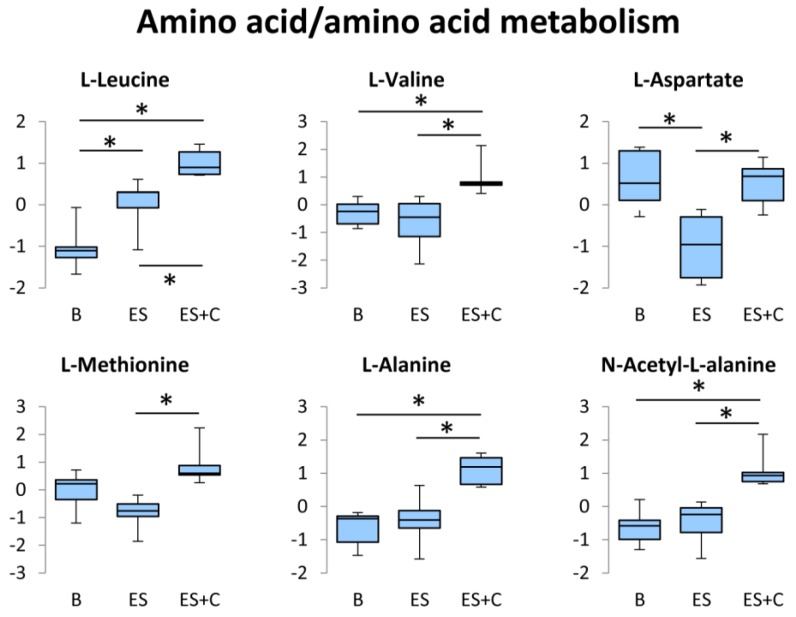
Box plots of the concentration variations of significantly altered metabolites in the amino acid/amino acid metabolism. * One-way ANOVA with Tukey’s post hoc test indicates a significant difference (FDR < 0.05) between groups.

**Figure 7:**
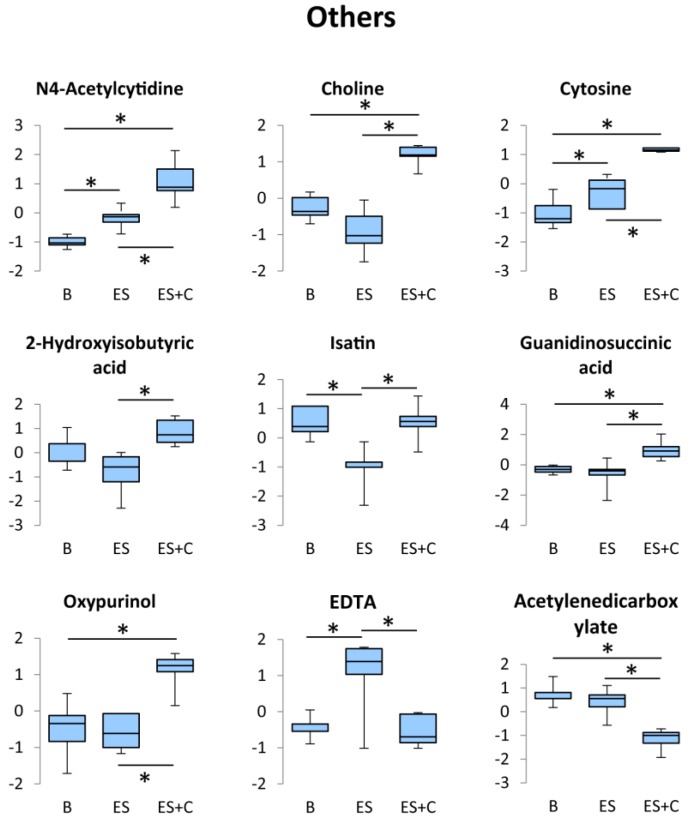
Box plots of the concentration variations of significantly altered metabolites in the other pathways. * One-way ANOVA with Tukey’s post hoc test indicates a significant difference (FDR < 0.05) between groups.

To identify differentially expressed metabolites among the three groups, SAM, a popular method employed in microarray data analysis [21], was used. SAM identified a total of 50 metabolites with FDR = 0.007 (Figure 2). Table 1 lists the identified compounds. HCA of the 50 metabolites showed that each group was tightly clustered and that the caffeine influenced the metabolite profiles of ES toward a high level of contents (Figure 3). Figure 4, Figure 5, Figure 6 and Figure 7 show the 23 metabolites that significantly changed between the ES and ES + C groups.

### 3.3. Pentose Phosphate Pathway/Nucleotide Synthesis Pathway

The pentose phosphate pathway is an alternative pathway to glycolysis [22]. It does not lead to ATP formation, but rather, produces ribose 5-phosphate. Intracellular ribose 5-phosphate concentration is an important determinant of rates of de novo purine synthesis [23]. The synthesis of purine nucleotides begins with ribose 5-phosphate and produces the first fully formed nucleotide, IMP. IMP is accumulated in contracted skeletal muscle during exercise and accounted for ATP re-synthesis during recovery phase from exercise [24]. Therefore, pentose phosphate pathway activation and subsequent nucleotide synthesis is suggested to be important for maintaining cellular energy during and following exercise.

In the present study, ES increased D-ribose 5-phophate levels, and caffeine further increased this effect (Figure 4), indicating that caffeine promotes exercise-induced activation of the pentose phosphate pathway in skeletal muscles. It was also found that caffeine in conjunction with ES increased IMP levels, as compared to ES alone (Figure 4). Taken together, the stimulation of the pentose phosphate pathway by caffeine may contribute to recovery from energy depletion following muscle contraction by promoting ATP re-synthesis from IMP. In our previous study, caffeine alleviated muscle fatigue during contraction [6]. Furthermore, it has been suggested that activation of the pentose phosphate pathway stimulates energy production by enhancing mitochondrial function [25]. These metabolic responses may contribute to a positive effect of caffeine on endurance performance.

A small proportion of IMP is converted to inosine and further to hypoxanthine, and hypoxanthine is transformed to xanthine, which is then subsequently converted to uric acid and excreted in the urine [26]. In the present study, hypoxanthine and xanthine levels were increased by caffeine treatment in the contracted muscle (Figure 4), supporting the caffeine-induced accumulation of IMP.

### 3.4. Acylcarnitine/Tricarboxylic Acid (TCA) Cycle

Acylcarnitines, which are esters of L-carnitine and fatty acyl-coenzyme A (CoA), are important intermediates in the transport of long-chain fatty acyl-CoA into the mitochondria [27]. Intramitochondrial acylcarnitine is converted back to carnitine and long-chain acyl-CoA by carnitine palmitoyltransferase 2, which then undergoes β-oxidation to produce acetyl-CoA. Acetyl-CoA is an essential intermediate metabolite that enters the TCA cycle and is oxidized to yield energy. When the production of short-chain acyl-CoAs exceeds TCA cycle flux, acetyl-CoA is converted to acetylcarnitine by carnitine acetyltransferase [28]. In the present study, caffeine treatment increased O-acetylcarnitine (C2) during skeletal muscle contraction (Figure 5), indicating that substrate catabolism during β-oxidation exceeds the capacity of acetyl-CoA utilization in the TCA cycle. 

Muscle contraction-induced AMPK activation inhibits acetyl-CoA carboxylase activity, leading to a decrease in malonyl CoA content [29]. Malonyl CoA is a potent inhibitor of CPT1, an enzyme that combines fatty acyl-CoA with carnitine for transport into the mitochondria for β-oxidation, and the decrease in malonyl CoA during muscle contraction contributes to the increase in absolute lipid oxidation [30]. In addition, it has been demonstrated that the increase in acetylcarinitine level during muscle contraction decreases the availability of free carnitine, a substrate of CPT1, results in low CPT1 activity [31]. Thus, the accumulation of acetylcarnitine within the skeletal muscle leads to a diminished supply of long-chain fatty acyl-CoA to β-oxidation [31]. In fact, an increase in acetylcarnitine was observed concomitantly with a decrease in long-chain fatty acid oxidation during exercise in humans [31]. In the present study, L-hexanoyl-carnitine (C6) was reduced by caffeine treatment (Figure 5), indicating that the increase in muscle acetylcarnitine level by caffeine might inhibit β-oxidation of long-chain fatty acyl-CoA, thereby leading to a decreased supply of short-chain (~C10) fatty acyl-CoA.

However, we found the accumulation of butyrylcarnitine (C4) following caffeine treatment (Figure 5). In accordance with this result, a previous study has demonstrated that the 10 min of treadmill exercise increased C4 acylcarnitine level in rat skeletal muscle [32]. The authors have suggested that the accumulation of C4 acylcarnitine was attributed to a greater utilization of branched chain amino acids (BCAA: leucine, isoleucine, and valine) [32]. We also found that caffeine treatment increased L-leucine and L-valine levels in contracted muscle (Figure 6). Therefore, it is suggested that the increase in butyrylcarnitine (C4) level originates from the amino acids metabolism.

Succinic acid is a key TCA cycle metabolite, the levels of which can be increased by both long-term exercise training as well as an individual bout of exercise [17,33]. In the present study, caffeine increased succinic acid levels in the contracted muscle (Figure 5). This result suggests that the TCA cycle is activated by caffeine treatment during muscle contraction. However, succinic acid level is reflected by the activity of succinate dehydrogenase, which catalyze succinic acid into fumarate in the TCA cycle. Therefore, to determine the effect of caffeine on the activity level of the TCA cycle during muscle contraction, succinate dehydrogenase level and/or another enzyme activity and metabolite levels need to be investigated.

**Figure 8:**
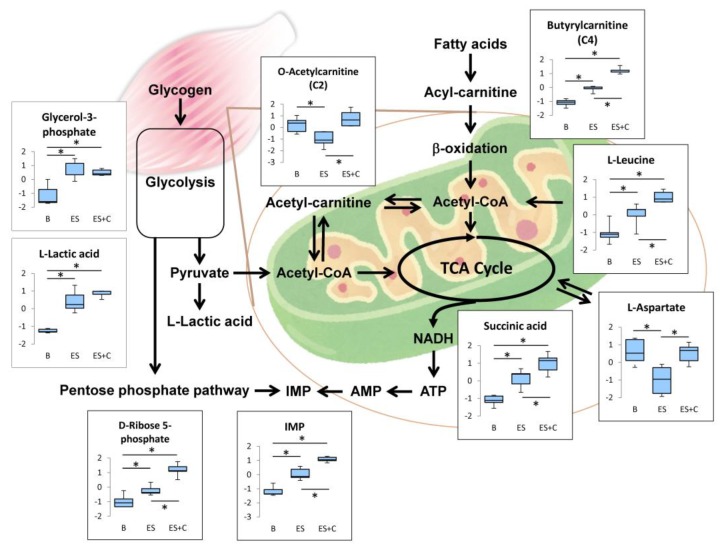
Schematic representation of metabolic pathway changes. * One-way ANOVA with Tukey’s post hoc test indicates a significant difference (FDR < 0.05) between groups.

In the present study, glycolysis was not affected by caffeine (Figure 8), suggesting that an increase in acetylcarnitine following caffeine treatment can be attributed to the acceleration of acetyl-CoA production from β-oxidation. Maintaining the acetylcarnitine recycling system is critical for muscle contractile performance and fatigue resistance [34]. Therefore, caffeine may stimulate the acetylcarnitine recycling system, thereby contributing to enhanced muscle endurance capacity. To assess this possibility, further study is required to measure the effects of caffeine on carnitine.

### 3.5. Amino Acid/Amino Acid Metabolism

Protein degradation and subsequent amino acid oxidation contribute slightly to energy supply during exercise as well as to glucose and fatty acid oxidation [35]. Six amino acids are metabolized in the skeletal muscle: BCAA (leucine, isoleucine, and valine), asparagine, aspartate, and glutamate [35]. Leucine can be converted to acetyl-CoA and oxidized in the TCA cycle [36]. The catabolic pathway of valine consists of several enzymatic steps and results in the formation of succinyl-CoA, a member of the TCA cycle. Disruption of BCAA metabolism in skeletal muscle impairs endurance capacity [37]. Thus, the supply of BCAA is considered to be an important factor for controlling exercise metabolism and endurance. In the present study, L-leucine and L-valine were higher in the ES + C group than in the ES group (Figure 6), indicating that these amino acids contribute to energy production of caffeine-treated muscle by incorporated into TCA intermediates.

Aspartate has been suggested to have an ergogenic potential [38]. Aspartate is converted to oxaloacetate by aspartate transaminase, which then enters the TCA cycle. A previous study has demonstrated that the administration of potassium-magnesium-aspartate increased the capacity for prolonged exercise in human [39]. This effect has been supported by other studies [38]. In the present study, caffeine suppressed L-aspartate reduction by ES (Figure 6), indicating that the caffeine-induced ergogenic effect may be partly attributed to aspartate preservation during muscle contraction. However, there are a number of negative findings related to aspartate’s ergogenic potential [38]. For example, in human volunteers neither the exerted force nor the endurance time increased after oral administration of potassium-magnesium-aspartate [40]. Therefore, further studies are required to clarify the relationship between aspartate and caffeine’s ergogenic potential.

Methionine is suggested to be transaminated and is also subjected to transulfuration in the skeletal muscle [41]. Although caffeine increased L-methionine levels (Figure 6) in the contracted muscle, the importance of methionine during exercise is poorly understood.

Alanine is formed from pyruvate and glutamate in the alanine aminotransferase reaction. Increase in skeletal muscle alanine is thought to be due to the enhanced availability of pyruvate and glutamate [42]. Alanine synthesized in the skeletal muscle is released into the blood and taken up by the liver, where it is reconverted into glucose via gluconeogenesis. In the present study, caffeine treatment increased L-alanine during muscle contraction (Figure 6). Considering that pyruvate and glutamate were not changed by caffeine stimulation (Table S1), this caffeine-induced increase in alanine may be attributed to protein degradation. The increase in alanine production may contribute to the increase in N-acetyl-L-alanine levels, which is generated from alanine by phenylalanine N-acetyltransferase, in caffeine and electrically stimulated muscle (Figure 6).

### 3.6. Others

N4-acetylcytidine, choline, cytosine, 2-hydroxyisobutyric acid, isatin, guanidinosuccinic acid, oxypurinol, ethylenediaminetetraacetic acid (EDTA), and acetylenedicarboxylate levels were significantly higher in the ES + C group than in the ES group (Figure 7). However, no previous studies have investigated the association between exercise and these metabolites. The functional significance of increased levels of these metabolites in the caffeine-stimulated group needs to be further investigated.

### 3.7. Limitations

Many reactions take place continuously within cells, so concentrations of metabolites are very dynamic. In the present study, we investigated metabolomic responses only 10 min after muscle contraction. Therefore, in a case where increased levels of metabolites are observed, two distinct mechanisms contribute to this observation: either increased production or decreased consumption. For further understanding of caffeine-mediated effects on metabolic changes during muscle contraction, time-course experiments should be conducted.

In this study, we used a concentration of caffeine at 3 mM, which would be toxic to humans [43]. Plasma concentration of caffeine after ingestion of 100 mg (1 cup of coffee) reaches approximately 5 to 10 μM [44], with less than 70 μM being the physiological concentrations [45]. Experiments using isolated skeletal muscle preparation have benefits of eliminating the effects of systemic confounders such as circulatory, humoral and neural factors, and of intestinal absorption of caffeine. Taking advantage of this point, a number of studies have unveiled the direct ergogenic properties of caffeine at the supraphysiological concentrations [10,46,47,48,49]. Our previous study have demonstrated that μM concentrations were enough to activate AMPK in vivo, but mM concentrations of caffeine were needed to activate AMPK in isolate rat skeletal muscle [5]. Therefore, we should be careful when comparing the results of in vitro and in vivo studies in terms of caffeine concentrations.

Caffeine is found in foods, beverages, and pharmaceuticals, and the most frequently consumed non-prescription drug. To date, many researchers have discussed the effect of caffeine on energy metabolism and our health [50,51,52]. However, no study has investigated the caffeine-mediated changes of metabolomic signatures in skeletal muscle and the other organs. Although the present study contributes to unveiling the effect of caffeine on metabolomic responses during muscle contraction condition, the effect of caffeine alone on skeletal muscle metabolism has not been cleared. Further studies are expected to examine the effect of caffeine on muscle’s non-contracted condition.

## 4. Conclusions

The present study reveals for the first time that caffeine influences metabolic responses induced by electrically stimulated muscle contraction in isolated rat skeletal muscles. A schematic representation of the metabolic changes induced by caffeine is shown in Figure 8. Many of these changes are related to energy metabolism. First, caffeine promotes contraction-induced activation of the pentose phosphate pathway and increases IMP production. Second, caffeine stimulates β-oxidation of fatty acyl-CoA, accompanied by increase in acyl-CoA, butylcarnitine and O-acetylcarnitine; however, it does not affect the glycolysis metabolites, glycerol-3-phosphate and L-lactic acid. Third, caffeine increases amino acids levels associated with energy production (L-leucine, L-valine, and L-aspartate). These metabolic alterations induced by caffeine suggest that caffeine accelerates contraction-induced metabolic activations and thereby contributes to muscle endurance performance and exercise benefits to health.

## Figures and Tables

**Table 1:** Significant features identified by SAM.

	Name	d Value	SD	*p* Value	q Value
1	Theobromine	5.7247	0.031023	0.0	0.0
2	Butyrylcarnitine (C4)	5.4855	0.055814	0.0	0.0
3	IMP	5.0676	0.10169	0.0	0.0
4	β-Aminoisobutyric acid	4.7254	0.14193	0.0	0.0
5	L-Lactic acid	4.6827	0.14713	0.0	0.0
6	Cytosine	4.4855	0.1717	5.4348 × 10^−5^	1.1445 × 10^−4^
7	D-Ribose 5-phosphate	4.4111	0.1812	5.4348 × 10^−5^	1.1445 × 10^−4^
8	D-Glyceraldehyde	4.3854	0.18452	5.4348 × 10^−5^	1.1445 × 10^−4^
9	Hypoxanthine	4.3489	0.18926	5.4348 × 10^−5^	1.1445 × 10^−4^
10	Maltotriose	4.2295	0.205	5.4348 × 10^−5^	1.1445 × 10^−4^
11	D-Glucosamine 6-phosphate	4.1269	0.21883	1.087 × 10^−4^	1.7607 × 10^−4^
12	Choline	4.1237	0.21927	1.087 × 10^−4^	1.7607 × 10^−4^
13	Succinic acid	4.0087	0.2351	1.087 × 10^−4^	1.7607 × 10^−4^
14	N4-Acetylcytidine	3.8995	0.25048	1.6304 × 10^−4^	2.4524 × 10^−4^
15	L-Glutamate	3.6907	0.28084	3.2609 × 10^−4^	4.2917 × 10^−4^
16	Acetylenedicarboxylate	3.6613	0.28523	3.2609 × 10^−4^	4.2917 × 10^−4^
17	L-Leucine	3.5719	0.29872	4.8913 × 10^−4^	5.7223 × 10^−4^
18	1-Myristoylglycerophosphocholine	3.5708	0.29889	4.8913 × 10^−4^	5.7223 × 10^−4^
19	L-Hexanoyl-carnitine (C6)	3.4897	0.31135	5.4348 × 10^−4^	6.0234 × 10^−4^
20	Serotonin	3.4538	0.31693	5.9783 × 10^−4^	6.2945 × 10^−4^
21	Threonate	3.4109	0.32367	7.0652 × 10^−4^	7.0847 × 10^−4^
22	Glycerol-3-phosphate	3.2018	0.35736	0.001413	0.0012937
23	2-Oxobutanoate	3.1997	0.35772	0.001413	0.0012937
24	ATP	3.009	0.38981	0.0021739	0.0018047
25	N-Acetyl-L-alanine	3.0039	0.3907	0.0021739	0.0018047
26	L-Alanine	2.9853	0.39391	0.0022283	0.0018047
27	Xanthine	2.9261	0.40421	0.0023913	0.001865
28	Imidazole lactic acid	2.913	0.40651	0.0025543	0.001921
29	N8-Acetylspermidine	2.8766	0.41292	0.002663	0.0019337
30	NADP	2.8527	0.41718	0.0029348	0.0020305
31	Oxypurinol	2.8504	0.41759	0.0029891	0.0020305
32	Taurine	2.7011	0.44464	0.0040761	0.0026823
33	O-Acetylcarnitine (C2)	2.6096	0.46169	0.0052174	0.0032651
34	Isatin	2.5882	0.46572	0.0052717	0.0032651
35	L-Fucose	2.5586	0.47133	0.0057065	0.0034334
36	Spermidine	2.5126	0.48016	0.0064674	0.0037831
37	1-Palmitoleoylglycerophosphocholine	2.4683	0.48872	0.0071739	0.0040357
38	GDP	2.4586	0.49062	0.0072826	0.0040357
39	Iminodiacetate	2.3301	0.51606	0.0096196	0.0051941
40	L-Methionine	2.3125	0.51962	0.010054	0.0052477
41	γ-Glu-leu	2.3104	0.52004	0.010217	0.0052477
42	Maleamate	2.2816	0.52587	0.010761	0.0052964
43	L-Aspartate	2.2673	0.52879	0.010815	0.0052964
44	Guanidinosuccinic acid	2.2214	0.53822	0.011793	0.0056442
45	L-Valine	2.169	0.54911	0.013478	0.0062447
46	CDP	2.1582	0.55136	0.013641	0.0062447
47	EDTA	2.134	0.55645	0.014511	0.0064614
48	2-Hydroxyisobutyric acid	2.126	0.55815	0.014728	0.0064614
49	Creatinine	2.1072	0.56212	0.015163	0.0065164
50	3-Methyl-2-oxobutyric acid	2.0385	0.57683	0.017065	0.0071872

## Supplementary Materials

The following are available online at https://www.mdpi.com/2072-6643/11/8/1819/s1, Table S1: List of detected metabolites.
