# Supplementary material for: The Effects of Caffeine on Metabolomic Responses to Muscle Contraction in Rat Skeletal Muscle
Source: Nutrients. 2019 Aug 7;11(8):1819. doi: 10.3390/nu11081819 (PMC6723980; doi:10.3390/nu11081819)
Supplement: Supplementary file 1 [file nutrients-11-01819-s001.pdf]

Table S1. List of detected metabolites

	Pathway Category	KEGG Number	Name	Ratio B vs. ES	Ratio B vs. ES+C
1	Acyl carnitine	C02571	O-Acetylcarnitine (C2)	0.77	1.11
2	Acyl carnitine	C02862	Butyrylcarnitine (C4)	2.27	3.93
3	Acyl carnitine	M00172	L-Hexanoyl-carnitine (C6)	0.77	0.34
4	Acyl carnitine	M00312	Octanoylcarnitine (C8)	0.00	0.00
5	Acyl carnitine	S00019	O-Dodecanoyl-L-carnitine (C12)	0.84	0.66
6	Acyl carnitine	S00020	O-Myristoyl-L-carnitine (C14)	0.40	0.32
7	Acyl carnitine	S00021	O-Stearoyl-L-carnitine (C18)	0.91	1.04
8	Alkaloids	C10172	Stachydrine	0.51	1.17
9	Amino acid	C00047	L-Lysine	1.02	1.35
10	Amino acid	C00062	L-Arginine	0.96	1.22
11	Amino acid	C00135	L-Histidine	0.88	1.21
12	Amino acid	C00037	Glycine	0.89	1.07
13	Amino acid	C00041	L-Alanine	1.08	1.59
14	Amino acid	C00065	L-Serine	0.89	1.13
15	Amino acid	C00183	L-Valine	0.92	1.28
16	Amino acid	C00188	L-Threonine	0.88	1.10
17	Amino acid	C00152	L-Asparagine	0.97	1.19
18	Amino acid	C00148	L-Proline	0.86	1.13
19	Amino acid	C00073	L-Methionine	0.83	1.21
20	Amino acid	C00064	L-Glutamine	0.85	0.87
21	Amino acid	C00025	L-Glutamate	0.63	0.71
22	Amino acid	C00078	L-Tryptophan	0.92	1.23
23	Amino acid	C00079	L-Phenylalanine	0.85	1.16
24	Amino acid	C00082	L-Tyrosine	0.87	1.18
25	Amino acid	C00049	L-Aspartate	0.69	1.00
26	Amino acid	C00123	L-Leucine	1.16	1.31
27	Amino acid metabolism	C01042	N-Acetyl-L-aspartate	0.92	0.99
28	Amino acid metabolism	M00296	Iminodiacetate	0.68	0.88
29	Amino acid metabolism	C02847	N-Acetyl-L-alanine	1.02	1.25
30	Arginin, Proline metabolism	C01924	Homoarginine	1.05	1.23
31	Arginin, Proline metabolism	C00791	Creatinine	0.79	0.88
32	Arginin, Proline metabolism	C03626	N-N-dimethylarginine	0.99	1.39
33	Arginin, Proline metabolism	M00001	N-alpha-Acetyl-L-arginine	1.22	1.22
34	Arginin, Proline metabolism	C03139	Guanidinosuccinic acid	0.92	1.29
35	Arginin, Proline metabolism	C01015	L-Hydroxyproline	0.57	0.79
36	Arginin, Proline metabolism	C00437	N-Acetylornithine	1.15	1.47
37	Ascorbate and Aldarate metabolism	C01620	Threonate	0.61	0.66
38	Butanoate metabolism	C01089	(R)-3-Hydroxybutanoate	0.96	1.31
39	Butanoate metabolism	C00497	(R)-Malate	1.01	1.10
40	Caffeine metabolism	C07480	Theobromine	2.45	11.48
41	Citrate cycle (TCA cycle)	C00417	cis-Aconitic acid	1.34	1.34
42	Citrate cycle (TCA cycle)	C00122	Fumarate	0.92	0.84
43	Citrate cycle (TCA cycle)	C00042	Succinic acid	1.92	2.54
44	Cofactors and Vitamins	C00864	(R)-Pantothenate	0.90	1.02
45	Cofactors and Vitamins	C00534	Pyridoxamine	0.81	0.95
46	Cofactors and Vitamins	C00378	Thiamine	1.45	1.70
47	Cofactors and Vitamins	C01004	Trigonelline	0.68	1.07
48	Cofactors and Vitamins	C00847	4-Pyridoxate	0.52	0.97
49	Cofactors and Vitamins	C00255	Riboflavin	0.81	0.76

50	Cysteine metabolism	C02989	L-Methionine S-oxide	0.82	1.07
51	Cysteine metabolism	C01234	1-Aminocyclopropane-1-carboxylate	1.02	1.14
52	Cysteine metabolism	C00170	5'-Methylthioadenosine	0.84	1.11
53	Dipeptide	M00236	Gly-Gly	0.92	1.05
54	Dipeptide	M00190	γ-Glu-leu	0.46	0.53
55	Dipeptide	C01419	L-Cysteinylglycine	0.96	1.39
56	Fatty Acid	C00803	Valerate	0.62	0.75
57	Fatty Acid	C01585	caproate (6:0)	0.74	0.73
58	Fatty Acid	C01571	Capric acid (10:0)	0.94	0.99
59	Fatty Acid	C08365	Ricinoleic acid (18:1-OH)	0.65	0.57
60	Fatty Acid	C08322	Myristoleate (14:1n5)	0.80	0.66
61	Fatty acid metabolism	C02678	Dodecanedioate	0.71	1.19
62	Glutathione metabolism	C00127	Oxidized glutathione	0.95	1.19
63	Glutathione metabolism	C00051	Glutathione	0.89	1.14
64	Glutathione metabolism	C01879	5-Oxoproline	0.92	0.81
65	Glycine, Serine metabolism	C00979	O-Acetyl-L-serine	1.20	0.91
66	Glycine, Serine metabolism	C00258	D-Glycerate	0.55	0.51
67	Glycine, Serine metabolism	C00114	Choline	0.80	1.44
68	Glycine, Serine metabolism	C00430	5-Aminolevulinate	0.81	1.17
69	Glycine, Serine metabolism	C01026	N,N-Dimethylglycine	0.98	1.73
70	Glycine, Serine metabolism	C00719	Betaine	0.91	1.00
71	Glycine, Serine metabolism	C00109	2-Oxobutanoate	0.79	0.75
72	Glycolysis	C00186	L-Lactic acid	7.04	8.49
73	Glycolysis	C00093	Glycerol-3-phosphate	3.15	2.98
74	Glycolysis	C05378	beta-D-Fructose 1,6-bisphosphate	3.32	0.82
75	Glycolysis	C00197	3-Phospho-D-glycerate	0.84	0.45
76	Glycolysis	C00074	Phosphoenolpyruvate	0.75	0.58
77	Glycolysis	C00022	Pyruvate	0.87	1.00
78	Histidine metabolism	C00388	Histamine	1.08	1.33
79	Histidine metabolism	M00007	3-Methylhistamine	1.16	1.13
80	Histidine metabolism	C01152	1-Methylhistidine	0.80	1.10
81	Histidine metabolism	C05828	Methylimidazoleacetic acid	0.79	1.96
82	Histidine metabolism	C02835	Imidazole-4-acetate	0.84	0.97
83	Histidine metabolism	C05568	Imidazole lactic acid	0.82	0.84
84	Lipid metabolism	C07599	Oxypurinol	0.96	1.81
85	Lipid metabolism	C01767	Carvone	0.68	0.55
86	Lipid(Glycerolphospholipid Metabolism)	C01996	Acetylcholine	1.05	1.12
87	Lysine metabolism	C00318	L-Carnitine	0.90	0.83
88	Lysine metabolism	C02727	N6-Acetyl-L-lysine	1.16	1.60
89	Lysine metabolism	C02427	Homocitrulline	0.89	1.09
90	Lysine metabolism	C00408	L-Pipecolate	0.95	1.02
91	Lyso PC	S00010	1-Myristoylglycerophosphocholine (14:0)	0.51	0.40
92	Lyso PC	S00012	1-Palmitoleoylglycerophosphocholine (16:1)	0.66	0.47
93	Lyso PC	C04102	1-Palmitoylglycerophosphocholine (16:0)	0.64	0.50
94	Nicotinate and Ncotinamide metabolism	C00003	NAD+	0.86	1.06
95	Nicotinate and Ncotinamide metabolism	C02918	1-Methylnicotinamide	1.00	0.97
96	Nicotinate and Ncotinamide metabolism	C00153	Nicotinamide	0.80	0.87

97	Nicotinate and Ncotinamide metabolism	C01596	Maleamate	0.81	0.89
98	Nicotinate and Ncotinamide metabolism	C15987	4-Methylaminobutyrate	0.86	1.04
99	Nicotinate and Ncotinamide metabolism	C00232	Succinate semialdehyde	0.73	1.02
100	Nicotinate and Ncotinamide metabolism	C00006	NADP+	1.43	1.69
101	Nicotinate and Ncotinamide metabolism	C00253	Nicotinate	1.03	1.07
102	Nucleotide metabolism	C02494	1-Methyladenosine	0.74	0.89
103	Nucleotide metabolism	M00261	N4-Acetylcytidine	1.66	2.53
104	Organic acid	C03761	3-Hydroxy-3-methylglutarate	0.86	0.89
105	Organic acid	C01984	Mandelate	0.65	0.90
106	Pentose phosphate pathway	C00085	D-Fructose 6-phosphate	0.65	0.60
107	Pentose phosphate pathway	C00117	D-Ribose 5-phosphate	1.68	2.97
108	Pentose phosphate pathway	C00279	D-Erythrose 4-phosphate	1.18	0.77
109	Pentose phosphate pathway	C00345	6-Phospho-D-gluconate	0.87	0.91
110	Phenylalanine Tyrosine metabolism	C05607	Phenyllactate	0.91	1.09
111	Phenylalanine Tyrosine metabolism	C05629	3-Phenylpropionate (hydrocinnamate)	0.76	0.72
112	Phosphonate and phosphinate metabolism	C00013	Pyrophosphate	0.92	0.87
113	Phosphonate and phosphinate metabolism	C00009	Phosphate	1.18	1.33
114	Polyamine	C00315	Spermidine	2.24	2.35
115	Polyamine	C01029	N8-Acetylspermidine	1.71	2.16
116	Prostaglandins	C00584	Prostaglandin E2	1.01	0.83
117	Purine metabolism	C00301	ADP-ribose	1.01	0.81
118	Purine metabolism	C00366	Urate	0.98	1.25
119	Purine metabolism	C00362	dGMP	0.68	0.80
120	Purine metabolism	C00008	ADP	0.74	0.85
121	Purine metabolism	C00130	IMP	4.14	6.53
122	Purine metabolism	C00002	ATP	0.60	0.60
123	Purine metabolism	C00035	GDP	0.69	0.80
124	Purine metabolism	C00044	GTP	0.74	0.86
125	Purine metabolism	C00054	Adenosine 3',5'-bisphosphate	0.38	0.43
126	Purine metabolism	C00212	Adenosine	0.29	0.53
127	Purine metabolism	C00262	Hypoxanthine	1.13	2.22
128	Purine metabolism	C00385	Xanthine	1.14	1.49
129	Purine metabolism	C00575	3',5'-Cyclic AMP	0.93	1.04
130	Purine metabolism	C00020	AMP	0.76	0.63
131	Purine metabolism	C00387	Guanosine	1.11	1.12
132	Purine metabolism	C00559	Deoxyadenosine	0.82	1.01
133	Pyrimidine metabolism	C00055	CMP	0.76	0.87
134	Pyrimidine metabolism	C00029	UDP-glucose	0.61	1.05
135	Pyrimidine metabolism	C00112	CDP	0.56	0.77
136	Pyrimidine metabolism	C00106	Uracil	1.22	1.11
137	Pyrimidine metabolism	C00105	UMP	0.61	0.82
138	Pyrimidine metabolism	M00023	5'-CMP	1.05	0.80
139	Pyrimidine metabolism	C00015	UDP	0.84	0.94
140	Pyrimidine metabolism	C00299	Uridine	1.15	1.16
141	Pyrimidine metabolism	C00380	Cytosine	1.26	1.65
142	Pyrimidine metabolism	C00906	5,6-Dihydrothymine	0.93	1.55
143	Pyrimidine metabolism	C00178	Thymine	0.89	0.80
144	Pyruvate metabolism	C03248	Acetylenedicarboxylate	0.97	0.82
145	Sphingolipids	C00346	Phosphoethanolamine	0.89	0.99
146	Steroids	M00242	α-Muricholic acid	0.66	1.48
147	Steroids	C03205	Cortexone	0.79	0.79

148	Steroids	C05463	Taurodeoxycholic acid	0.69	0.82
149	Steroids	C00410	Progesterone	0.76	0.78
150	Sugar	C00310	D-Xylulose	0.93	1.08
151	Sugar	C00221	β-D-Glucose	0.95	1.06
152	Sugar	C01835	Maltotriose	0.55	0.42
153	Sugar	C00392	D-Mannitol	0.78	0.82
154	Sugar	C01019	L-Fucose	0.78	0.83
155	Sugar	C03383	D-Galactono-1,4-lactone	1.07	1.10
156	Sugar metabolism	C00043	UDP-N-acetylglucosamine	0.97	1.01
157	Sugar metabolism	C00577	D-Glyceraldehyde	2.13	2.21
158	Sugar metabolism	C00352	D-Glucosamine 6-phosphate	2.95	2.87
159	Taurine and hypotaurine metabolism	C00519	Hypotaurine	0.77	0.97
160	Taurine and hypotaurine metabolism	C00245	Taurine	0.86	0.85
161	Tryptophan metabolism	C01717	Kynurenic acid	1.22	1.65
162	Tryptophan metabolism	C08304	Gramine	0.79	0.72
163	Tryptophan metabolism	C00780	Serotonin	2.13	2.95
164	Unclassified	C01807	Mucate	0.82	1.21
165	Unclassified	C01205	β-Aminoisobutyric acid	0.59	0.57
166	Unclassified	M00246	EDTA	2.14	0.94
167	Unclassified	M00012	2-Hydroxyisobutyric acid	0.82	1.18
168	Unclassified	C11129	Isatin	0.52	1.00
169	Unclassified	C00571	Cyclohexylamine	0.93	0.72
170	Unclassified	M00022	(±)-1,2-Diphenylethylenediamine	0.87	0.93
171	Unclassified	C01517	Naproxen	0.61	0.82
172	Urea Cycle	C00270	N-Acetylneuraminate	0.99	1.18
173	Urea Cycle	C00019	S-Adenosyl-L-Methionine	1.22	1.35
174	Urea Cycle	C01035	4-Guanidinobutanoate	0.85	1.05
175	Urea Cycle	C00300	Creatine	0.87	1.02
176	Urea Cycle	C00213	Sarcosine	0.81	0.83
177	Urea Cycle	C00327	L-Citrulline	0.87	1.02
178	Valine, leucine and isoleucine metabolism	C02612	Citramalic acid	0.86	1.13
179	Valine, leucine and isoleucine metabolism	C00141	3-methyl-2-oxobutyric acid	1.67	1.80
180	Valine, leucine and isoleucine metabolism	C00671	3-Methyl-2-oxovaleric acid	0.82	0.64
181	Valine, leucine and isoleucine metabolism	C02710	N-Acetyl-L-leucine	0.83	0.73
182	β-Alanine metabolism	C00386	Carnosine	0.81	0.96
183	β-Alanine metabolism	C01262	Anserine	0.82	1.02
184	β-Alanine metabolism	C00099	β-Alanine	0.84	0.85
